# Selective cytotoxic effect of *Plantago lanceolata* L. against breast cancer cells

**DOI:** 10.1186/s43046-019-0010-3

**Published:** 2019-12-30

**Authors:** Khulood M. Alsaraf, Maeda H. Mohammad, Ahmed Majeed Al-Shammari, Ibrahim S. Abbas

**Affiliations:** 1 Pharmacy Department, Al-Esraa University College, Baghdad, Iraq; 2 Experimental Therapy Department, Iraqi Center for Cancer and Medical Genetic Research, Mustansiriyah University, Baghdad, Iraq; 3 Department of Pharmacognosy and Medicinal Plants, College of Pharmacy, Mustansiriyah University, Baghdad, Iraq

**Keywords:** Cytotoxicity, HPLC analysis, Flavonoids, Iraq, Clonogenic assay

## Abstract

**Background:**

*Plantago lanceolata* L. is used in Iraqi folklore medicine to treat injuries, and its extract is prescribed by some herbalists for cancer patients. This research aimed to evaluate the effect of *P. lanceolata* leaf extract on breast cancer cell lines in vitro and to identify its active compounds. Crystal violet viability assay was used to determine the cytotoxicity of methanolic *P. lanceolata* leaf extract against various breast cancer cell lines. MCF7, AMJ13, MDAMB, and CAL51 human breast cancer cells were treated with different concentrations of the extract for 72 h. The morphology of the treated cells was examined under a phase-contrast inverted microscope. The clonogenic ability was assessed through a clonogenic assay. High-performance liquid chromatography (HPLC) analysis was performed to measure the concentrations of phenols and flavonoids in the extract.

**Results:**

The methanolic *P. lanceolata* leaf extract significantly inhibited the proliferation of triple-negative CAL51 cells but showed minor effect on the other breast cancer cells. In addition, at high doses, it induced cytopathic morphological changes. The clonogenic assay showed low colony formation in the exposed cells, especially CAL51 cells. Furthermore, HPLC study revealed that the methanolic extract contained important flavonoid glycosides, especially rutin, myricetin quercetin, and kaempferol.

**Conclusions:**

*P. lanceolata* leaf extract selectively inhibited the proliferation of CAL51 triple-negative breast cancer cells and showed minor effect on the other breast cancer cells types studied. Thus, this study showed *P. lanceolata* as a possible natural source of selective anti-triple-negative breast cancer drugs.

## Background

Cancer is a complex disease that is difficult to be treated [1]. Cancer incidence has increased globally, and especially in Iraq, it has increased owing to several factors related to environmental pollution associated with several years of conflicts [2]. Breast malignant tumors are the second leading cause of mortality among Iraqi women [3]. However, conventional cancer treatments, such as chemotherapy, radiation, targeted therapy, and immunotherapy, have harmful side effects. Therefore, herbal medicine has been considered a useful alternative to the currently existing therapies [4]. Herbal medicine is widely used for treating several types of cancers because it has fewer side effects than conventional cancer therapeutics [5].

In Iraq, ribwort (*Plantago lanceolata* L.) is used for injury treatment [6], and its extract is prescribed by some herbalists to cancer patients. This plant is one of the medicinally important plants officially registered in the British pharmacopeia. Ribwort, also known as narrow leaf plantain, is rich in phytochemical compounds [7]. Ribwort is originally native to Europe and temperate areas of Asia, but it has been cultivated in all temperate regions of the world [8]. *P. lanceolata* leaves are erect, straight, and sheltered with soft minute hairs; they reach up to approximately 17 in. long and taper at the base into a slender petiole. The flowers are condensed spikes on the top of the stalks; the flowers consist of a small corolla, four sepals, and two stamens [9]. This plant contains major secondary metabolites including mucilages (6%), tannins (more than 5%) [10], and phenyl propanoids [11], with verbascoside as a major constituent [12], as well as iridoid glycosides [13], mainly aucubin and cutalpol. It also contains minor secondary metabolites, such as flavonoids [14], coumarins, volatile compounds [15], and saponin. Global studies showed that ribwort seeds have external uses for treating skin inflammation and healing wounds [16]. It is found that crude extracts of Plantago leaves have anti-proliferative effect on MCF7 breast cancer cell line [17] and against prostate cancer cell lines [18]. However, the antitumor effect of this plant has not been much studied. Thus, this study aimed to identify the active compounds of *P. lanceolata* leaf extract and evaluate its antitumor effect on cancer cell lines in vitro.

## Methods

### Collection of plant samples

*P. lanceolata* leaves were collected from a Usefia region south Baghdad at middle of Iraq. The plants were confirmed to be uninfected and healthy, and authenticated by the National Herbarium of Iraq who undertook the formal identification of the plant material used in our study, and a specimen of this material has been deposited in a National Herbarium of Iraq under the number (56432). The leaves were washed by clean tap water and distilled water to eliminate dust and other foreign particles, air-dried at room temperature, ground in a blender, and then weighed.

### Plant extraction

Plant leaves (100 g) were ground into fine powder using a stainless-steel grinder and then extracted in 70% ethanol (200 ml) overnight. The fraction extracted by ethanol was isolated by using a muslin cloth and sterile filtered through a Whatman filter (No.02) paper. The filtered extract was then concentrated in a rotary evaporator.

### Analysis of phenol and flavonoid content in *P. lanceolata* leaf extract

Detection of flavonoid was performed using a high-performance liquid chromatography (HPLC) system from Shimadzu, Japan, following previously described methods [19] at the Department of Chemistry, Ministry of Science and Technology. The column used was Shimpack C-18 (particle size of 3 μm; 50 × 4.6 mm 1.D). The mobile phase was 0.1% phosphoric acid-acetonitrile (52:24, v\v). UV detection was set at 285 nm. The flow rate was 1.5 ml/min, whereas the temperature was 25 °C. The concentration of each component was measured quantitatively by comparing the detected peak area of the samples with that of a standard according to the following equation:

$$ \mathrm{Sample}\ \mathrm{concentration}=\frac{\mathrm{area}\ \mathrm{of}\ \mathrm{the}\ \mathrm{sample}}{\mathrm{area}\ \mathrm{of}\ \mathrm{the}\ \mathrm{Standard}}\times \mathrm{standard}\ \mathrm{concentration}\times \mathrm{dilution}\ \mathrm{factor} $$

### Maintenance of cell cultures

The human breast cancer cell lines AMJ13 [20], MCF7, MDAMB, and CAL51, as well as mouse embryo fibroblasts (MEF) were supplied by Cell Bank Unit. AMJ13 cells were cultured in RPMI-1640 medium (USbiological, USA) supplemented with 10% fetal bovine serum (FBS) (Capricorn Scientific, Germany), 100 units/mL penicillin, and 100 μg/mL streptomycin. MCF7, MDAB, and CAL51 cells were cultured in Minimum Essential medium (MEM) (USbiological) supplemented with 10% FBS (Capricorn- Scientific, Germany), 100 μg/mL streptomycin, and 100 units/mL penicillin. The cells were incubated at 37 °C in a humidified environment and 5% CO_2_.

### Cytotoxicity assays

Crystal violet cell viability assay was employed to measure the cytotoxic effect of the plant extract. Human breast cancer cell lines (MDAMB, AMJ13, MCF7, and CAL51 cells) and normal mouse embryonic cells (MEF) were seeded at a density of 7000 cells/well in 96-well plates (Santa Cruz Biotechnology, USA). After 24 h of incubation or after a confluent monolayer was formed, the cells were treated with *P. lanceolata* leaf extract at 2-fold dilutions (4000, 2000, 1000, 500, 250, 125, 62.5, 31.25 μg/ml to 15 μg/ml) in culture media. The assay was performed in triplicate. Cell viability was determined after 72 h of treatment by cell staining with 50 μl of crystal violet (Sigma Aldrich, USA) followed by incubation at 37 °C for 2 h. The stain was aspirated, and PBS was used to wash the wells. A microplate reader (Biochrom, UK) was used to measure the absorbance at 492 nm. Results of the assay were shown as percentage of proliferation relative to control cells [21].

### Morphology and quantitative image analyses

The treated and untreated cells were photographed at four haphazardly selected cultured fields using an inverted light microscope at × 200 magnification (Leica Microsystems, Germany) and digital color camera (Leica-microsystems, Germany). The images were examined using the ImageJ software (http://rsb.info.nih.gov/ij/). For statistical analysis, quantitative measurement of each picture was performed in triplicate. Percentage (%) of cells stained by crystal violet were calculated [22].

### Clonogenic assay

The cells were seeded at a density of 5 × 10^5^ cells/mL in 6-well tissue culture plates and incubated until confluency. Next, the cells were exposed to *P. lanceolata* leaf extract at different concentrations. The cells were then stained with crystal violet stain and examined under an inverted microscope [23].

### Statistical analysis

The data are presented as means ± standard error of the mean. One-way analysis of variance was used for data comparison between treatment groups. Differences in data were considered statistically significant at *P* < 0.05. For this analysis, we used the GraphPad Prism 6 software (GraphPad Software, Inc. San Diego, California).

## Results

### Chemical structure analysis

**Fig. 1:**
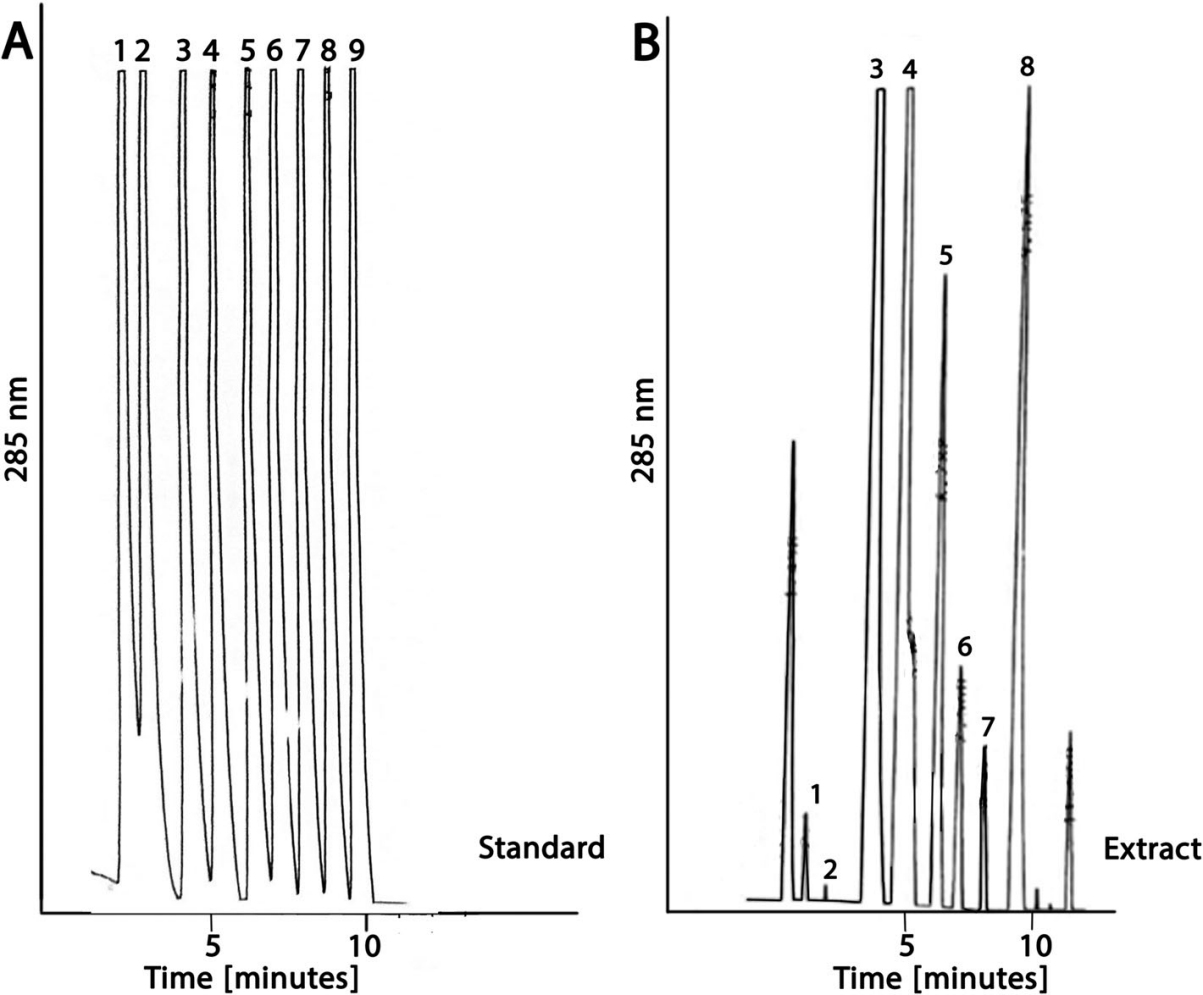
HPLC chromatogram of *P. lanceolata* leaf extract. **a** HPLC trace standards. **b** P. lanceolata leaves ethanol extract monitored at 285 nm. Peak identification: 1, protoctecuic acid; 2, cafeic acid; 3, ferulic acid; 4, sinapic acid; 5, O-cumaric; 6, rutin; 7, myricetin; 8, quercetin

The results of HPLC analysis are presented in Table 1. The results showed that *P. lanceolata* leaf extract contained important flavonoid glycosides, especially rutin, myricetin quercetin, and kaempferol at concentrations of 13.37 μg, 15.27 μg, 16.99 μg, and 15.37 μg, respectively. The percentage of these compounds was higher than that of the standard. Thus, the activity of *P. lanceolata* leaf extract may be attributed to these bioactive compounds (Fig. 1).

**Table 1:** Bioactive compounds of *P. lanceolata* leaf extract, identified by HPLC

Seq	Standard	Retention time (minutes)	Area	Concentration μg/ml	Seq	Sample	Retention time (minutes)	Area	Concentration μg/ml
1	Protocatecuic acid	2.198	576685	12.10	1	Protoctecuic acid	2.152	65876	3.88
2	Cafeic acid	2.957	572594	12.02	2	Cafeic acid	2.898	43719	5.23
3	Ferulic acid	4.373	545481	11.45	3	Ferulic acid	4.352	9.ooo55	7.84
4	Sinapic asid	5.44	445366	9.35	4	Sinapic acid	5.423	6.20378	9.78
5	O-cumaric	6.672	441940	9.28	5	O-cumaric	6.782	284530	12.26
6	Rutin	7.60	584451	12.27	6	Rutin	7.568	168223	13.37
7	Myricetin	8.597	566220	11.88	7	Myricetin	8.475	103305	15.27
8	Quercetion	9.537	487263	10.23	8	Quercetin	9.425	438903	16.99
9	Kaempferol	10.443	544223	11.42	9	Kaempferol	8.425	57964	12.16

### Cytotoxicity assay

**Fig. 2:**
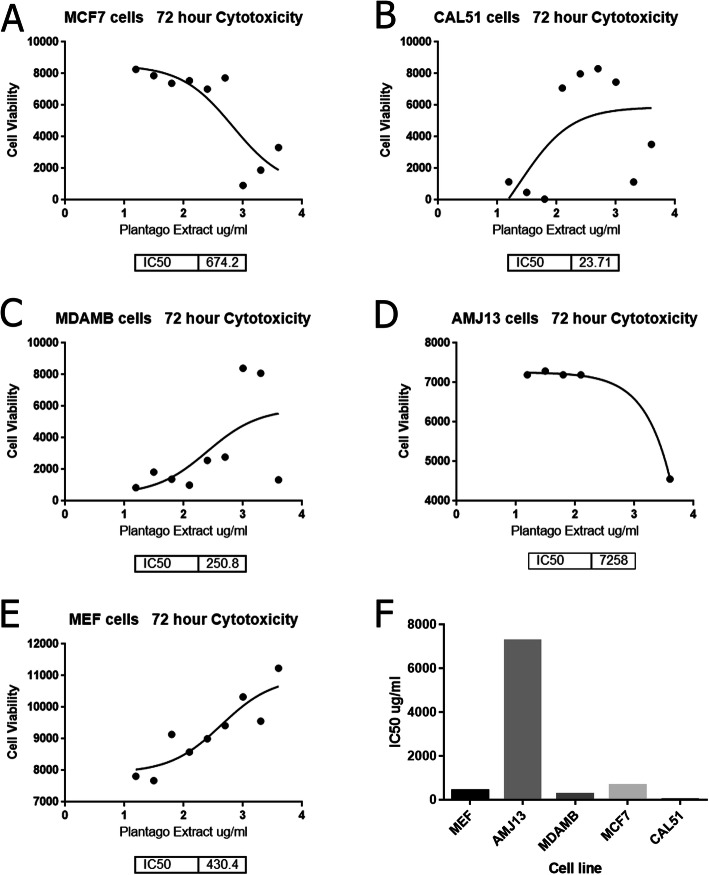
The *P. lanceolata* leaf extract exhibited cytotoxicity against breast cancer cell lines, with different IC50 values of **a** 674.2 μg/ml for MCF7 cells, **b** 23.7 μg/ml for CAL51 cells, **c** 250.8 μg/ml for MDAMB cells, and **d** 7.2 mg/ml for AMJ13 cells, as well as against **e** normal embryonic fibroblast cells, with the IC50 value of 430 μg/ml. **f** Comparative study of IC50 values showed that CAL51 cells were the most sensitive to the extract

**Fig. 3:**
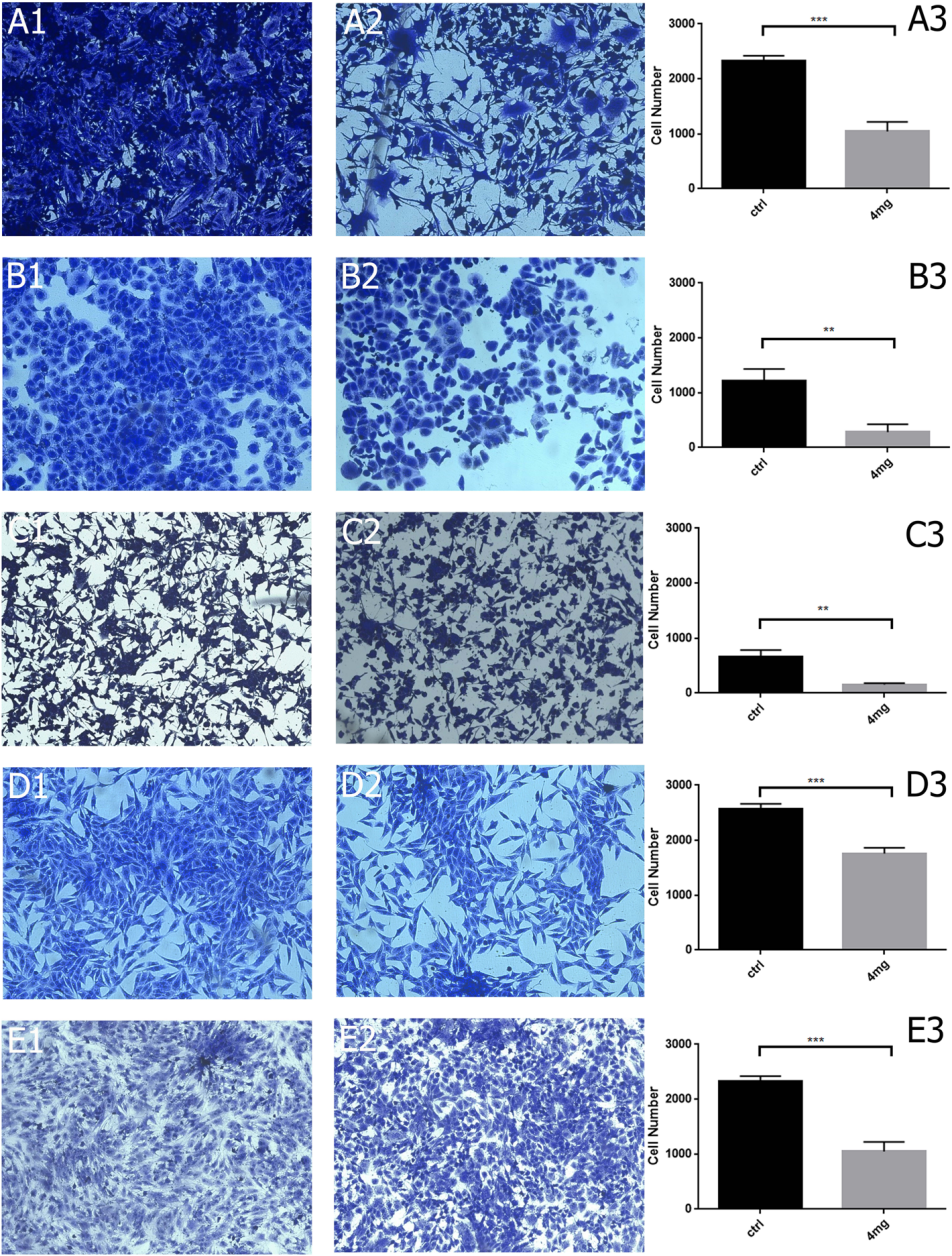
Cytomorphology and quantitative image analysis of the treated and control cells. **a** MCF-7 cells: A1, control cells; A2, treated cells; A3, graph showing the efficacy of high-dose extract. **b** CAL51 cells: B1, control; B2, treated; B3, graph the efficacy of high-dose extract. **c** MDAMB cells: C1, control cells; C2, treated cells; C3, graph showing the efficacy of high-dose extract. **d** AMJ13 cells: D1, control; D2, treated extract; D3, graph showing the efficacy of high-dose extract. **e** MEF cells: E1, control cells; E2, treated cells; E3, graph showing that high-dose extract was cytotoxic to normal cells; × 400 magnification; crystal violet staining

The *P. lanceolata* leaf extract showed the most potent cytotoxic effects against the triple-negative cancer cell line CAL51, with an IC50 value of 23.7 μg/ml (Fig. 2b). In contrast, the extract showed no noticeable cytotoxic effect against MCF7, AMJ13, and MDAMB cells, with extremely high IC50 values for these cells: 250 μg/ml for MDAMB cells, 674 μg/mL for MCF7 cells, and 7200 μg/ml for AMJ13 cells. For comparison, the IC50 value of the extract for normal embryonic fibroblast cells was 430 μg/mL (Fig. 2). The cells treated with the extract showed decreased cell number owing to cell detachment, as observed in most of the observed fields. Furthermore, these cells showed condensed nuclei, indicating early apoptosis in the treated cells compared to untreated cells; this finding was observed in the cells treated with the highest concentration of extract. On the contrary, untreated cancer cells continued to proliferate to form monolayers. Early apoptotic cells with condensed nuclei and darker stain as well as lightly stained normal cells are shown in Fig. 3.

### Clonogenic assay

**Fig. 4:**
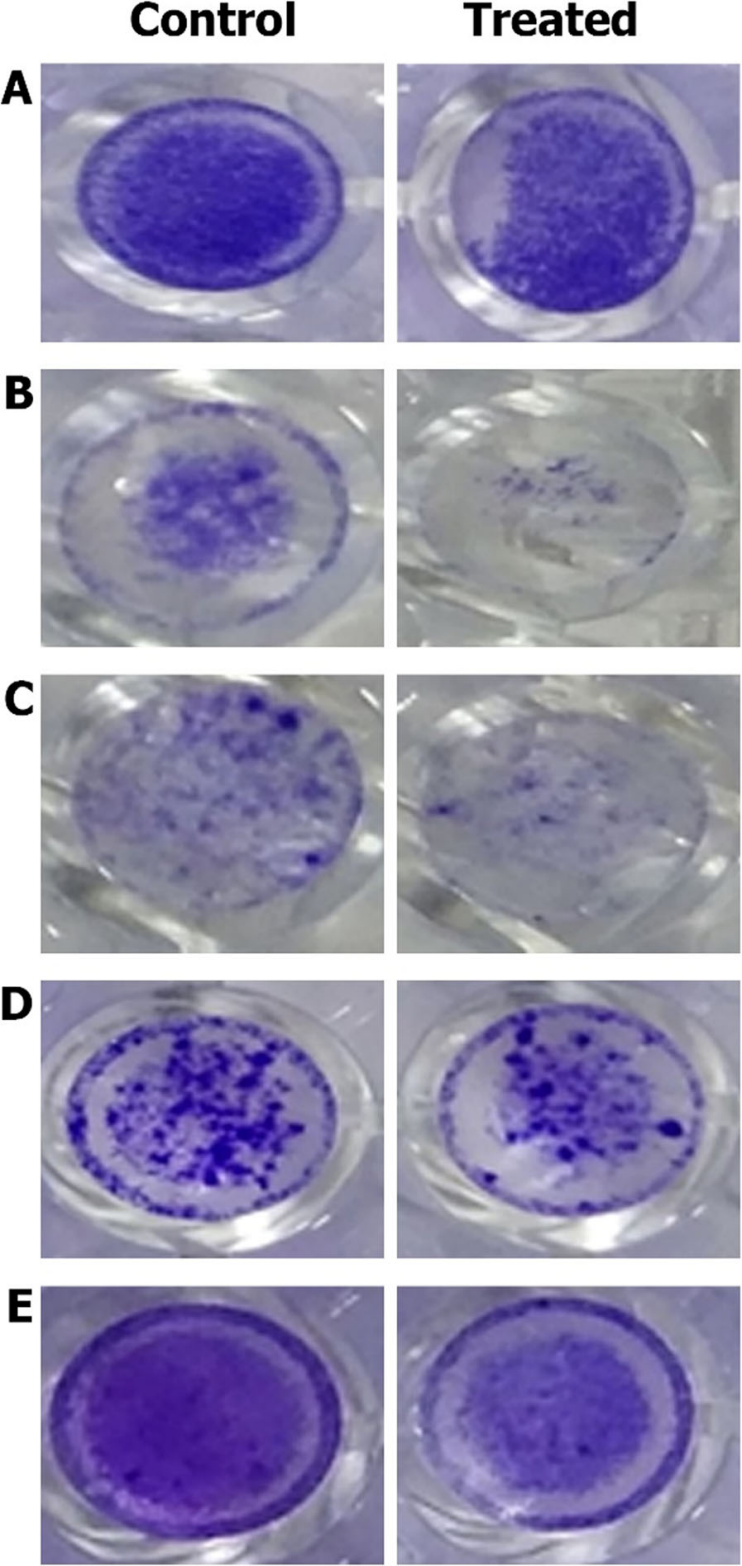
Colony-forming ability of cell cultures was investigated by clonogenic assay to assess the cytotoxicity of *P. lanceolata* leaf extract. **a** MCF-7 cells treated with high-dose (4 mg/ml) extract, compared to the control cells. **b** CAL51 cells showing the efficacy of high-dose (4 mg/ml) extract in suppressing colony forming ability of the CAL51 cells. **c** MDAMB, showing a high dose of extract in compare to 4 mg/ml. **d** AMJ13, showing a high dose of extract was less effective. **e** MEF, control untreated cells showed complete cells growth to confluency in compare to 4 mg/ml treatment. The cells were stained with crystal violet

More experiments were performed to assess the cytotoxic activity of *P. lanceolata* leaf extract at different concentrations against several breast cancer cell lines (MCF-7, CAL51, MDAMB, and AMJ13 cells) and normal embryonic cells (MEF cells) using clonogenic assay. The efficacy of the extracts in inhibiting colony formation of the tested cells was determined. *P. lanceolata* leaf extract at a high concentration of 4 mg/mL showed high efficacy in inhibiting colony formation of CAL51 cells but showed low efficacy in inhibiting that of the other breast cancer cells. The extract at the same concentration showed less effect on other breast cancer cells and on normal MEF colony forming when we used the same concentration (Fig. 4). These findings suggested that *P. lanceolata* leaf extract selectively suppressed the proliferation of CAL51 triple-negative breast cancer cells.

## Discussion

The search of natural sources for cancer therapeutics has brought promising results thus far. Active compounds have been identified and isolated for use in cancer therapy. Several species of *Plantago* have been described as remedies for treating tumors [24]. In this study, flavonoids were identified by HPLC as major constituents of *P. lanceolata* leaf extract. Flavonoids are a group of compounds with different phenolic structures and known for their health benefits. There are over 4000 types of flavonoids; several of them are responsible for the color of fruits, flowers, and leaves [25]. The oxygen-derived free radical-scavenging activity of flavonoids has been shown to lead to anticarcinogenic effects [26]. Antioxidative effect is the defining feature of nearly every group of flavonoids. Flavones and catechins are flavonoids with the ability to protect the body from reactive oxygen species-induced damage. Cellular organelles and components can be damaged by reactive oxygen species and other free radicals, which are generated as a result of exogenous stimuli or oxygen metabolism [27]. Increased production of reactive oxygen species leads to consumption and exhaustion of endogenous free radical-scavenging compounds. Flavonoids may exert additive effect on these endogenous free radical-scavenging compounds [28].

The results of cytotoxicity and clonogenic assays in the present study revealed that *P. lanceolata* leaf extract exhibited antiproliferative effect and cytotoxicity against breast carcinoma cell lines, especially CAL51 triple-negative breast cancer cells. The cytotoxic effect of the extract may be attributed to its flavonoid content, namely flavone and luteolin-7-O-β-glucoside [29]. Luteolin-7-O-β-glucoside is a promising anticancer molecule with anti-breast adenocarcinoma activity [24]. Another team [30] found that *P. lanceolata* extract is effective against MCF7 cell line and the IC50 value is 142.78 μg/ml while our study found that IC50 on MCF7 cells is 674.2 μg/ml and the difference may attributed to the difference in extraction method, but it still higher than required for CAL51 breast cancer cells. The IC50 value on prostate cancer cell lines PC-3 and Du-145 was about 300 μg/ml [18]. These results are the same range of our results but not for the AMJ13 which was very resistant, while CAL51 was very sensitive as IC50 value was 23.71 μg/ml.

## Conclusions

This study reported, for the first time, that *P. lanceolata* leaf extract exerted selective antiproliferative effect against CAL51 triple-negative breast cancer cells. Thus, this extract was potential for development as an anticancer agent for triple-negative breast cancer patients.

## Data Availability

The datasets used and/or analyzed during the current study are available from the corresponding author on reasonable request.

## Abbreviations

FBS: Fetal bovine serum
HPLC: High-performance liquid chromatography
IC50: Inhibitory concentrations 50%
MEF: Mouse embryo fibroblasts

## References

[1] O’Connor CM, Adams JU, Fairman J. Essentials of cell biology, vol. 1. Cambridge, MA: NPG Education; 2010.

[2] Al-Shammari AM. Environmental pollutions associated to conflicts in Iraq and related health problems. Rev Environ Health. 2016;31(2):245–50. 26512425 10.1515/reveh-2015-0024

[3] (Iraq) MoH. Iraq Cancer Registry 2015. Baghdad, Iraq: Ministry of Health (Iraq); 2015.

[4] Alsabah AS, Abd AH, Al-Shammari AM. Cytotoxicity of Xanthium strumarium against breast cancer cell lines. J Global Pharma Technol. 2018;10(3):767–76.

[5] Sahoo N, Manchikanti P, Dey SH. Herbal drug patenting in India: IP potential. J Ethnobiol Ethnomed. 2011;137(1):289–97. 10.1016/j.jep.2011.05.022 21640810

[6] Ahmed HM. Ethnopharmacobotanical study on the medicinal plants used by herbalists in Sulaymaniyah Province, Kurdistan, Iraq. J Ethnobiol Ethnomed. 2016;12:8. 26821541 10.1186/s13002-016-0081-3 PMC4730727

[7] Grigore A, Bubueanu C, Pirvu L, Ionita L, Toba G. Plantago lanceolata L. Crops–source of valuable raw material for various industrial applications. Scientific Papers-Series A, Agronomy. 2015;58:207–14.

[8] Jacke D, Toensmeier E. Edible Forest Gardens, Volume II: ecological design and practice for temperate-climate permaculture: Chelsea Green Publishing; 2005.

[9] Hasan AK, Ibrahim SA, Amani AT, Monther FM. Determination, isolation, and identification of aucubin and verbascoside in the leaves of Iraqi Plantago lancoleta L. using different detecting methodS. Int J Pharm Pharm Sci. 2018;11(2).

[10] Maksyutina N. Hydroxycinnamic acids of Plantago major and Pl. lanceolata. Chem Nat Compd. 1971;7(6):795.

[11] Andary C, Motte-Florac M, Gargadennec A, Wylde R, Heitz A. Les esters caféiques du genre Plantago. Identification et valeur chimiotaxinomique. Pl Med Phytotherap. 1988;22(1):17–22.

[12] Murai M, Tamayama Y, Nishibe S. Phenylethanoids in the herb of Plantago lanceolata and inhibitory effect on arachidonic acid-induced mouse ear edema 1. Planta Med. 1995;61(05):479–80. 7480214 10.1055/s-2006-958143

[13] Handjieva N, Saadi H, Evstatieva L. Iridoid Glueosides from Plantago altissima L., Plantago lanceolata L., Plantago atrata Hoppe and Plantago argentea Chaix. Zeitschrift für Naturforschung C. 1991;46(9-10):963–5.

[14] Wichtl M. Herbal drugs and phytopharmaceuticals: a handbook for practice on a scientific basis: Medpharm GmbH Scientific Publishers; 2004.

[15] Fons F, Rapior S, Gargadennec A, Andary C, Bessière J-M. Volatile components of Plantago lanceolata (Plantaginaceae). Acta Bot Gallica. 1998;145(4):265–9.

[16] Núñez Guillén ME, da Silva Emim JA, Souccar C, Lapa AJ. Analgesic and anti-inflammatory activities of the aqueous extract of Plantago major L. Int J Pharmacogn. 1997;35(2):99–104.

[17] Daştan SD, Daştan T, Cetinkaya S, Ateşşahin D, Karan T. Evaluation of in vitro anticancer effect of Plantago major L. and Plantago lanceolata L. leaf extracts from Sivas. Cumhuriyet Üniversitesi Sağlık Bilimleri Enstitüsü Dergisi. 2016;1(1):7–14.

[18] Asadi-Samani M, Rafieian-Kopaei M, Lorigooini Z, Shirzad H. A screening of growth inhibitory activity of Iranian medicinal plants on prostate cancer cell lines. BioMedicine. 2018;8(2):8. 29806586 10.1051/bmdcn/2018080208 PMC5992925

[19] Kashams A, Hamza MA, Abbas IS. Effect of humic and salicylic acids on oil yield and flavonoid glycoside of safflowers (Carthamus tinctorius L.) as medicinal plants grown in Iraq. Int J Pharm Sci Res. 2018;9(5):2100–4.

[20] Al-Shammari AM, Alshami MA, Umran MA, Almukhtar AA, Yaseen NY, Raad K, et al. Establishment and characterization of a receptor-negative, hormone-nonresponsive breast cancer cell line from an Iraqi patient. Breast Cancer: Targets Ther. 2015;7:223–30. 10.2147/BCTT.S74509 PMC4536763 26300657

[21] Al-Shammari AM, Salman MI, Saihood YD, Yaseen NY, Raed K, Shaker HK, et al. In vitro synergistic enhancement of Newcastle Disease Virus to 5-fluorouracil cytotoxicity against tumor cells. Biomedicines. 2016;4(1):3. 28536371 10.3390/biomedicines4010003 PMC5344244

[22] Al-Shammari AM, Syhood Y, Al-Khafaji AS. Use of low-power He-Ne laser therapy to accelerate regeneration processes of injured sciatic nerve in rabbit. Egypt J Neurol Psychiatr Neurosurg. 2019;55(1):1. 30679899 10.1186/s41983-018-0047-6 PMC6320753

[23] Jabir MS, Taha AA, Sahib UI, Taqi ZJ, Al-Shammari AM, Salman AS. Novel of nano delivery system for Linalool loaded on gold nanoparticles conjugated with CALNN peptide for application in drug uptake and induction of cell death on breast cancer cell line. Mater Sci Eng C. 2019;94:949–64. 10.1016/j.msec.2018.10.014 30423784

[24] Gálvez M. Martı, x, n-Cordero C, López-Lázaro M, Cortés F, et al. Cytotoxic effect of Plantago spp. on cancer cell lines. J Ethnopharmacol. 2003;88(2):125–30. 12963131 10.1016/s0378-8741(03)00192-2

[25] de Groot H, Rauen U. Tissue injury by reactive oxygen species and the protective effects of flavonoids. Fundam Clin Pharmacol. 1998;12(3):249–55. 9646056 10.1111/j.1472-8206.1998.tb00951.x

[26] Panche AN, Diwan AD, Chandra SR. Flavonoids: an overview. J Nutr Sci. 2016;5:e47-e. 28620474 10.1017/jns.2016.41 PMC5465813

[27] Jung W, Chung I, Kim S, Kim M, Ahmad A, Praveen N. In vitro antioxidant activity, total phenolics and flavonoids from celery (Apium graveolens) leaves. J Med Plant Res. 2011;5(32):7022–30.

[28] Garcia-Orozco KD, Sanchez-Paz A, Aispuro-Hernandez E, Gomez-Jimenez S, Lopez-Zavala A, Araujo-Bernal S, et al. Gene expression and protein levels of thioredoxin in the gills from the whiteleg shrimp (Litopenaeus vannamei) infected with two different viruses: The WSSV or IHHNV. Fish Shellfish Immunol. 2012;32(6):1141–7. 22465360 10.1016/j.fsi.2012.03.020

[29] Pettit GR, Hoard MS, Doubek DL, Schmidt JM, Pettit RK, Tackett LP, et al. Antineoplastic agents 338. The cancer cell growth inhibitory. Constituents of Terminalia arjuna (Combretaceae). J Ethnopharmacol. 1996;53(2):57–63. 8844460 10.1016/S0378-8741(96)01421-3

[30] Beara IN, Lesjak MM, Orčić DZ, Simin NĐ, Četojević-Simin DD, Božin BN, et al. Comparative analysis of phenolic profile, antioxidant, anti-inflammatory and cytotoxic activity of two closely-related Plantain species: Plantago altissima L. and Plantago lanceolata L. LWT - Food Science and Technology. 2012;47(1):64–70.

